# Transcriptional gene silencing requires dedicated interaction between HP1 protein Chp2 and chromatin remodeler Mit1

**DOI:** 10.1101/gad.320440.118

**Published:** 2019-05-01

**Authors:** Karoline Leopold, Alessandro Stirpe, Thomas Schalch

**Affiliations:** 1 Department of Molecular Biology, Faculty of Science, Sciences III, University of Geneva, CH-1211 Geneva 4, Switzerland;; 2 Leicester Institute for Structural and Chemical Biology, Department of Molecular and Cell Biology, University of Leicester, Leicester LE1 9HN, United Kingdom

**Keywords:** heterochromatin, nucleosome remodeling, *S. pombe*, HP1 proteins, recruitment, X-ray crystallography, protein interactions

## Abstract

Heterochromatin protein 1 (HP1) proteins are key factors of eukaryotic heterochromatin that coordinate chromatin compaction and transcriptional gene silencing. Through their multivalency they act as adaptors between histone H3 Lys9 di/trimethyl marks in chromatin and effector complexes that bind to the HP1 chromoshadow domain. Most organisms encode for multiple HP1 isoforms and the molecular mechanisms that underpin their diverse functions in genome regulation remain poorly understood. In fission yeast, the two HP1 proteins Chp2 and Swi6 assume distinct roles and Chp2 is tightly associated with the nucleosome remodeling and deacetylation complex SHREC. Here we show that Chp2 directly engages the SHREC nucleosome remodeler subunit Mit1. The crystal structure of the interaction interface reveals an extraordinarily extensive and specific interaction between the chromoshadow domain of Chp2 and the N terminus of Mit1. The integrity of this interface is critical for high affinity binding and for heterochromatin formation. Comparison with Swi6 shows that the Chp2–Mit1 interface is highly selective and thereby provides the molecular basis for the functional specialization of an HP1 isoform.

## Abstract (precis)

Here, Leopold et al. investigated the molecular mechanisms which control the diverse functions of multiple HP1 isoforms in genome regulation. Their results show how a dedicated and extensive molecular interaction between a specific HP1 protein and the gene silencing machinery drives functional specialization of HP1 isoforms.

Eukaryotic genomes are highly organized and division into euchromatic and heterochromatic compartments is crucial for the correct execution of gene expression programs, for establishment of chromosomal structures such as telomeres and centromeres and for protection of the genome from parasitic genetic elements (Grewal and Jia 2007; Schalch 2017).

Heterochromatin protein 1 (HP1) was first discovered as a marker for constitutive heterochromatin at centromeres and chromocenters in *Drosophila,* and since then HP1 and its isoforms have been found to play various critical roles in heterochromatin and euchromatin (Eissenberg and Elgin 2014). HP1 proteins are prime examples of epigenetic reader proteins as they feature a chromodomain (CD) that binds the H3K9 di/trimethyl modification on histones, a hallmark of heterochromatic regions (Bannister et al. 2001; Lachner et al. 2001; Nakayama et al. 2001). The second conserved domain of HP1 proteins is their chromoshadow domain (CSD), which serves to dimerize and bind client proteins (Platero et al. 1995; Ye et al. 1997). Recruitment of chromatin effector proteins by HP1 is highly conserved and uses a linear peptide motif in the HP1 binding partners of the consensus sequence PxVxL, which inserts between the C termini of the HP1 CSD dimer (Smothers and Henikoff 2000; Thiru et al. 2004). The two conserved domains are connected by a flexible hinge region and thus HP1 proteins are proposed to act as adapters that bridge histone marks and effector proteins and thereby determine chromatin function (Li et al. 2002). Dimerization also renders HP1 proteins bivalent for H3K9 methyl marks, which governs HP1 association dynamics and influences compaction of heterochromatin (Azzaz et al. 2014; Kilic et al. 2015; Hiragami-Hamada et al. 2016). HP1 proteins are further regulated by posttranslational modifications (LeRoy et al. 2009; Nishibuchi et al. 2014; Kilic et al. 2015), and phosphorylation has been proposed to regulate segregation of heterochromatin by HP1-driven phase separation (Larson et al. 2017; Strom et al. 2017).

Most organisms have multiple HP1 isoforms, including mammals (HP1α, HP1β, and HP1γ), *Xenopus laevis* (HP1α and HP1γ), *Drosophila* (HP1, HP1b, HP1c, Rhino, and HP1E), *Caenorhabditis elegans* (HPL-1 and HPL-2), and fission yeast (Swi6 and Chp2) (Vermaak et al. 2005; Lomberk et al. 2006). Despite their similar architecture and sequence they are remarkably different. For example, different HP1 isoforms associate with different sets of client proteins (Motamedi et al. 2008; Fischer et al. 2009; Vermeulen et al. 2010). The different isoforms also often have nonoverlapping roles in genome regulation (Minc et al. 1999; Smothers and Henikoff 2001; Vakoc et al. 2005), and it remains poorly understood how diversification of function is established since they bind the same histone mark and the same motif in client proteins. Here we investigate the mechanism underlying HP1 diversification in the fission yeast *Schizosaccharomyces pombe,* where the nonoverlapping functions of the two HP1 isoforms in heterochromatin formation is well established.

Gene silencing in the *S. pombe* system relies on an interplay between RNA interference (RNAi) and chromatin-associated processes (Martienssen and Moazed 2015). Small RNAs are produced from pericentromeric heterochromatin loci and guide the RNAi-induced transcriptional gene silencing complex (RITS) to nascent transcripts, which are subsequently degraded by the RNAi machinery (Volpe et al. 2002; Verdel et al. 2004; Shimada et al. 2016). Concomitantly, RITS recruits the H3K9 methyltransferase Clr4 to heterochromatic loci (Zhang et al. 2008; Bayne et al. 2010), and deposition of the H3K9 methyl marks provides chromatin binding sites for the HP1 proteins Swi6 and Chp2. These proteins cannot complement each other's function in the regulation of transcription at heterochromatic loci (Sadaie et al. 2008), and they are found to be part of nonoverlapping complexes (Motamedi et al. 2008; Fischer et al. 2009). While Swi6 is expressed at relatively high levels, Chp2's expression levels are much lower (Sadaie et al. 2008). In contrast to Swi6, which binds a wide range of client proteins, Chp2 is predominantly associated with the Snf2/HDAC repressor complex (SHREC) that belongs to the family of nucleosome remodeling and deacetylation complexes (NuRDs).

SHREC consists of the nucleosome remodeler Mit1, the histone deacetylase Clr3 and the MBD-like protein Clr2, which are all connected by the scaffolding subunit Clr1 (Sugiyama et al. 2007; Motamedi et al. 2008; Fischer et al. 2009; Job et al. 2016). The previous work has also established a close functional and biochemical relationship between Chp2 and the Mit1 remodeler, which recruits the SHREC complex to heterochromatin. However, how Chp2 achieves the specific recruitment of the SHREC complex is not understood.

Here we show that Chp2 binds the N terminus of Mit1, and that an extensive interface between Chp2 and Mit1 provides a high-affinity interaction that is required for recruitment of Mit1 to heterochromatin and silencing of gene expression. Our structural and functional analysis provides insight into how an isoform-specific HP1 complex forms and how it contributes to heterochromatin function.

## Results

### Chp2 interacts with the N terminus of Mit1 to repress transcription

**Figure 1.**
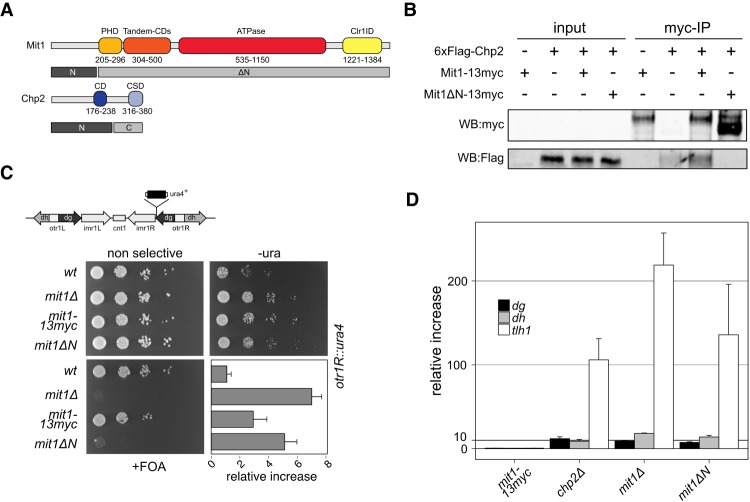
The Mit1 N terminus mediates Chp2 interaction and is essential for heterochromatin silencing. (*A*) Domain diagram of the Mit1 remodeler and the HP1 homologue Chp2. Gray bars correspond to fragments used in *B*–*D* and Supplemental Figure S1A,B. (PHD) Plant homeodomain finger; (CD) chromodomain; (CSD) chromoshadow domain; (Clr1ID) Clr1-interacting domain; (N) N terminus; (C) C terminus. (*B*) Coimmunoprecipitation of endogenously tagged Mit1-13myc and 6xFlag-Chp2. (*C*) Serial dilution growth assays of wild-type *mit1+* and *mit1ΔN* mutant. Strains were assessed for growth on PMG media, on PMG-ura to monitor *otr1R::ura4*+ expression, and PMG + FOA to monitor silencing of *otr1R::ura4*+. Changes in steady-state transcript levels in mutant strains relative to wild-type cells were measured by quantitative real-time RT-qPCR for the *otr1::ura4* reporter (*C*) and for centromeric *dg/dh* repeats and *tlh1* transcripts at telomeres (*D*). *act1* was used as internal standard for all measurements and standard errors were calculated from three independent biological experiments.

Chp2 recruits the SHREC complex to elicit transcriptional gene silencing in heterochromatic regions (Motamedi et al. 2008; Fischer et al. 2009). These mass spectrometry experiments suggested that the interaction occurs between Chp2 and either the SHREC scaffold Clr1 or the chromatin remodeler Mit1. To identify the molecular interface that underlies Chp2-mediated recruitment of SHREC we chose insect-cell based coexpression of Chp2 with Mit1 or Clr1. Because pull-down experiments of Chp2 with fragments of the Clr1 N terminus did not yield a complex, we focused on the chromatin remodeler Mit1 and its individual domains (Fig. 1A). Coexpression of a tertiary complex consisting of Chp2, Mit1, and Clr1's Mit1 interaction domain (MID) yielded a stable biochemical entity corresponding to the remodeler module of SHREC (Supplemental Fig. S1A). By testing various domain deletions of Mit1 for complex formation we found that the N terminus of Mit1, which does not harbor any known domains, was necessary and sufficient for mediating the Chp2 interaction in the heterologous insect cell system. Deletion of residues 61–200, which are predicted to be unstructured, lead to loss of the interaction, while the mutation of two LxVxL motifs in this region (Mit1V20F, Mit1V72F) (Supplemental Fig. S1A, lane N2F) has no effect on complex formation. These results suggested that Mit1 and Chp2 might deviate from a canonical PxVxL-mediated interaction. To confirm and further characterize complex formation we subjected Mit1 and Chp2 to a yeast-two-hybrid assay (Supplemental Fig. S1B). The yeast-two-hybrid results showed that the Mit1 N terminus and the Chp2CSD are each necessary and sufficient for interaction. Under stringent selection on quadruple drop-out medium (QDO) Chp2 full length protein is required to sustain growth. This raises the possibility that Mit1 interacts with the CSD as well as the CD, the hinge region or the N terminus of Chp2.

To investigate the role of the Mit1 N terminus under physiological conditions we endogenously tagged Mit1 with a C-terminal 13-myc tag and deleted the N-terminal 200 amino acids (*mit1ΔN*) (Supplemental Fig. S1C). Coimmunoprecipitation experiments in a 6xFlag-Chp2 background showed complete loss of Chp2 interaction in the *mit1ΔN* mutant (Fig. 1B; Supplemental Fig. S1D). We therefore conclude that Chp2 forms a complex with Mit1 by binding to its N-terminal domain.

We next subjected the *mit1ΔN* strain to comparative growth assays in a *otr1R::ura4* background that allows assessment of heterochromatin integrity in the pericentromeric region of chromosome 1 in order to test the functional relevance of the Chp2–Mit1 interaction. In a *ura4Δ* background the expression levels of the *ura4* marker genes can be monitored by increased growth of cells on medium lacking uracil. In contrast, medium containing the drug 5-fluorouracil (FOA) inhibits growth of cells that fail to silence *ura4*, because its gene product converts FOA into fluorodeoxyuridine, which is toxic to the cells. Similar to a complete deletion of *mit1*+, deletion of the Mit1 N terminus leads to loss of silencing of the *otr1R::ura4* reporter gene (Fig. 1C). Thus, full length Mit1 is critical for heterochromatin formation. To analyze the function of the Mit1 N terminus at endogenous loci we measured centromeric and telomeric transcript levels by RT-qPCR and compared them to wild-type cells (Fig. 1D). We found five to 15-fold elevated levels of centromeric transcripts in the *mit1ΔN* mutant when compared to wild type. This is similar to the changes observed at the *otr1R::ura4* reporter (Fig. 1C). Even though the myc-tagged wild-type allele shows a small defect at the *otr1R::ura4* reporter it is fully functional at endogenous loci. As expected from previous observations (Sugiyama et al. 2007; Motamedi et al. 2008), the centromeric transcript changes for *mit1* mutants are moderate when compared to *clr4Δ* (Supplemental Fig. S1E). In our experiments telomeric transcripts show a stronger relative change in silencing between wild-type and mutant levels than the centromeric repeats, which might be due to the extremely low levels of transcripts in wild-type cells and consequently large fold-changes upon loss of silencing. Nevertheless, at all loci heterochromatic transcript levels in *mit1ΔN* cells are very similar to the ones observed for *chp2Δ* or *mit1Δ*. These results suggest that an intact physical connection between Chp2 and Mit1 is essential for mediating their function in heterochromatin formation.

### Crystal structure of the Chp2–Mit1 interface reveals extensive interaction

**Figure 2.**
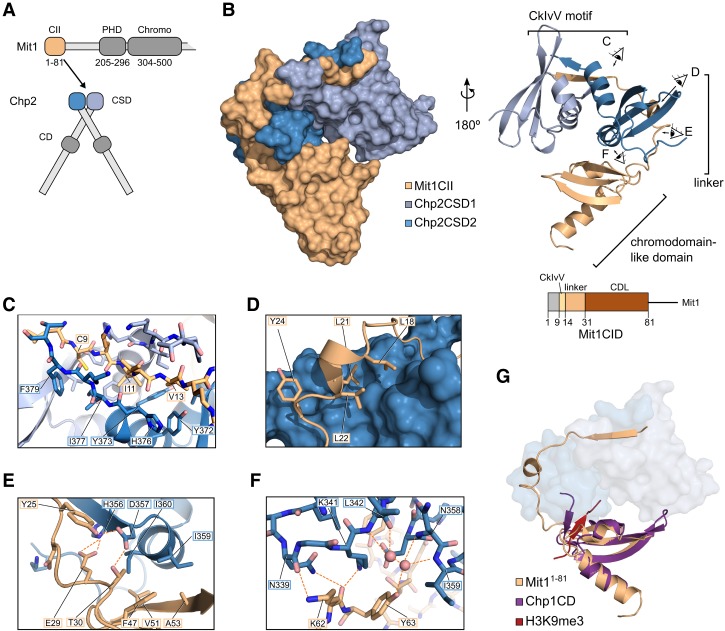
Crystal structure of the Chp2–Mit1 complex reveals extensive interface. (*A*) Schematic of the minimal complex between the N-terminal 81 residues of Mit1 and the CSDs of Chp2 based on limited proteolysis (Supplemental Fig. S1A). (*B*) Surface and cartoon representation of the Chp2–Mit1 crystal structure. Eye symbols with letters indicate viewing angles for corresponding details panels *C*–*F*. (*C*–*F*) Close-up views of the Chp2–Mit1 crystal structure colored as in (*B*). (*C*) Binding of the CkIvV motif to the groove formed by the Chp2CSD dimer. (*D*) Hydrophobic interactions of the linker region of Mit1 with the surface of Chp2CSD2. (*E*) Hydrogen bonding network between Mit1 linker domain and Chp2CSD2. (*F*) Water-mediated Mit1CDL–Chp2CSD2 interaction interface. (*G*) Superposition of the Mit1CDL domain with the CD of Chp1 bound to a H3K9 trimethyl peptide (PDBID: 3G7L, RMSD = 1.38 Å).

To understand how Chp2 recognizes the N terminus of Mit1 we decided to determine the atomic structure of the interaction interface. Limited proteolysis of a complex consisting of the Chp2CSD and the N-terminal 300 residues of Mit1 revealed a protected Mit1 fragment corresponding to residues 1–81, which we will refer to as the Chp2 interaction interface (CII) (Fig. 2A; Supplemental Fig. S2A). This Mit1 fragment formed a stable complex with Chp2 (Supplemental Fig. S2B), which readily crystallized and allowed us to determine the structure by molecular replacement at a resolution of 1.6 Å (Table 1). Electron density is well defined for the two CSD copies of Chp2, and we observed an equally well defined density for residues 8–81 of the Mit1CII (Supplemental Fig. S2C).

**Table 1.**
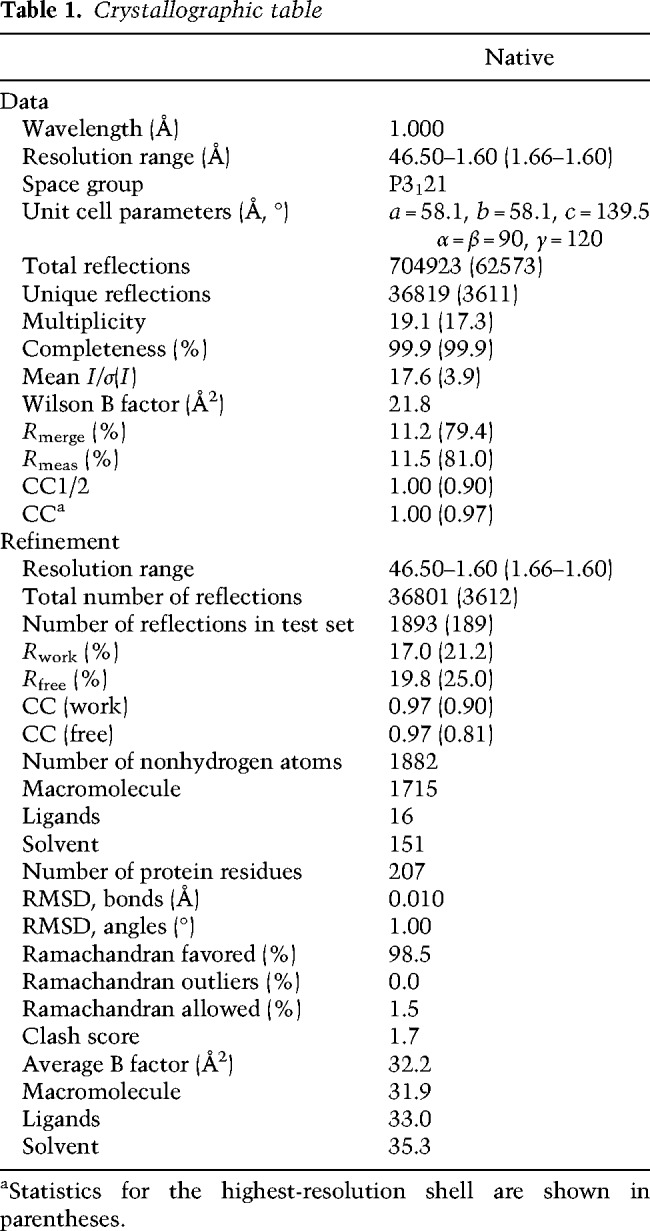
Crystallographic table

The structure of this minimal Chp2–Mit1 complex revealed a 2:1 stoichiometry with one Mit1 molecule bound to one Chp2CSD dimer (Fig. 2B). Mit1CII occupies the canonical peptide-binding groove at the CSD dimerization interface and, intriguingly, it wraps around one of the Chp2CSDs and engages an extensive interaction interface. The structure allows for the unambiguous identification of residues 9–13 of Mit1 in the CSD dimerization grove. These residues correspond to the sequence CKIVV with I11 occupying the center position on the symmetry axis of the CSD dimer (position 0 when numbered according to Thiru et al. 2004) (Fig. 2C). The central I11 as well as C9 at position −2 and V13 at position +2 are buried inside hydrophobic pockets at the bottom of the binding groove, while K and V at positions ±1 are exposed to the solvent. The binding groove is lined by two symmetric sets of residues comprising Y372, Y373, H376, I377, and F379 from both Chp2 protomers. The central I11 is a large hydrophobic side chain, and when compared to HP1-CAF1 (Thiru et al. 2004), a canonical HP1-PxVxL complex, Chp2 accommodates the extra bulk of the isoleucine by a side chain rotation of Y373 such that the aromatic face of the tyrosine residue packs against the methyl groups of I11 (Supplemental Fig. S2D). The outer pocket where the side chains of C9 and V13 are found is formed by Y372 and H376 from one protomer and F379 and I377 from the other protomer. This pocket is relatively spacious and easily accommodates the cysteine and valine residues, but without strong complementarity or formation of specific hydrogen bonds. Thus, the structure suggests that the Chp2–Mit1 equivalent of the HP1–PxVxL interaction is a CkIvV motif that binds in a well-defined manner to the Chp2CSD dimer interface due to spacious hydrophobic binding pockets and the hydrogen bonds established by β-sheet formation.

### The Chp2–Mit interface includes a cryptic Mit1 domain with CD fold

The crystal structure reveals that the interface between Mit1 and Chp2 includes extensive interactions that go far beyond the classical PxVxL binding groove at the CSD dimer interface. These additional interactions are provided by residues downstream of the CkIvV motif, which are bound to the surface of Chp2CSD2 (Fig. 2B). The first stretch of residues, which we refer to as the linker region, assumes an extended configuration and corresponds to Mit1 positions 15–31. It contains a short 3^10^ helix formed by a hydrophobic stretch between residues 18–22. The three leucines on this helix all fit complementary pockets in the Chp2 surface (Fig. 2D). The structure also shows that the V20 of the previously tested LxVxL motifs (Supplemental Fig. S1A) is on the surface and can accommodate a mutation to phenylalanine comfortably (the same is true for V72). After these hydrophobic interactions follows a series of polar and acidic residues, which form a hydrogen-bonding network with residues H356 and D357 of Chp2 (Fig. 2E; Supplemental Fig. S2E,F). Chp2H356 engages the side chain of Mit1E29 in a bifurcated hydrogen bond and Chp2D357 establishes one hydrogen bond with Mit1Y25 and one with Mit1T30. Due to these hydrophobic and polar interactions the entire Mit1 linker region is tightly associated with the Chp2 surface and is therefore expected to play a significant role in mediating the interaction between Chp2 and Mit1.

The linker region is followed by a folded domain, easily recognizable as a CD fold with close resemblance to the Chp1 CD (Fig. 2G; Schalch et al. 2009). The domain assumes the typical arrangement of a warped three-stranded β sheet that packs against a C-terminal α-helix. However, the Mit1 CD-like (CDL) domain lacks an aromatic cage and the binding groove for a methyl-lysine histone peptide. Instead, the peptide binding groove of the domain is occupied by Chp2CSD2 (Fig. 2F,G), with Chp2I360 occupying the space of the H3K9 trimethyl group when compared with Chp1.

The contacts between Chp2 and the Mit1CDL domain extend the interface established by the hydrogen bonding network in the linker region (Supplemental Fig. S2F). Key residues in the CDL are Mit1K62 and Mit1Y63. The Mit1K62 side chain is involved in a hydrogen bond to the backbone oxygen of Chp2N339 and reciprocally the side chain group of Chp2K341 hydrogen-bonds to the backbone oxygen of Mit1K62. Mit1Y63 is involved in a water-mediated hydrogen-bonding network that connects it to backbone atoms of Chp2N358, Chp2I359, and Chp2L342 (Fig. 2F). Furthermore, we found hydrophobic contacts between Chp2F319 and Mit1A49 at the edge of the CDL, as well as in the hydrophobic core of the Mit1CDL–Chp2CSD interface that is formed by Mit1F47, Mit1V51, Mit1A53, Chp2I360, and Chp2I359 (Supplemental Fig. S2F).

The Chp2–Mit1 structure reveals an extensive interface that buries 3354 Å^2^ of surface area involving the CkIvV motif, linker region and CDL domain (CkIvV motif: 1465 Å^2^, Linker: 1131 Å^2^, CDL: 750 Å^2^). Each of them provides a significant number of hydrogen bonds and hydrophobic interactions. In contrast to the CkIvV motif, which displays little side chain specificity, the Mit1 linker and CDL domain engage in numerous hydrogen-bonding interactions. The structure predicts that all three parts are required for full binding affinity and that linker and CDL provide the extra specificity of Mit1 for the Chp2CSD.

### The extended Chp2–Mit1 interface is required for high affinity interaction

**Figure 3.**
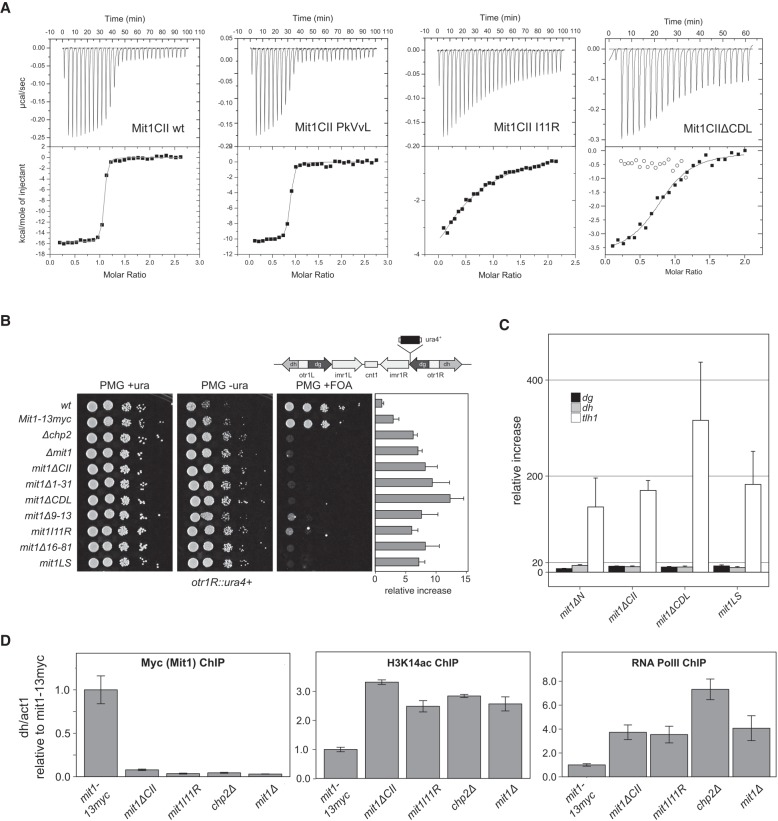
Extended interaction between Chp2 and Mit1 is required for heterochromatin silencing. (*A*) ITC heat rates and fits for titration of Chp2CSD with StrepSumo-tagged Mit1CII wild-type and indicated mutant proteins. Open circles represent StrepSumo-tag only control. See Table 2 for fitted parameters. (*B*) Comparative growth assays for Mit1 mutants in an *otr1R::ura4*+ background with the corresponding transcript levels of the *otr1R::ura4* reporter displayed as horizontal bars measured by quantitative real-time RT-PCR. RT-qPCR levels for wild type, *mit1-13myc*, *Δchp2,* and *Δmit1* from Figure 1D are shown for comparison (*C*) Changes in steady-state transcript levels in mutant strains relative to wild-type cells were determined by RT-qPCR for centromeric *dg/dh* repeats and *tlh1* transcripts at telomeres. *mit1ΔN* from Figure 1D is shown for comparison. (*D*) Chromatin immunoprecipitation (ChIP) for indicated mutant strains against Mit1-13myc, the H3K14ac histone mark and for RNA polymerase II. *act1* was used as internal standard for all measurements and standard errors were calculated from three independent biological experiments.

To characterize the biophysical properties of the Chp2–Mit1 interaction we subjected the complex to analysis by isothermal calorimetry (ITC) measurements. Figure 3A shows that combination of wild-type Mit1CII and Chp2CSD results in a exothermic binding reaction, revealing a tight interaction between Chp2 and Mit1 with a *K*_d_ of 2.6 nM (Table 2). As Chp2 is predicted to bind canonical PxVxL motifs (Thiru et al. 2004) we tested binding of a mutant Mit1 with engineered PkVvL motif instead of CkIvV, and obtained a *K*_d_ of 8.2 nM. These data show that despite its nonconsensus sequence the CkIvV motif binds at least equally well to the Chp2CSD dimer as a canonical PxVxL motif.

**Table 2.**
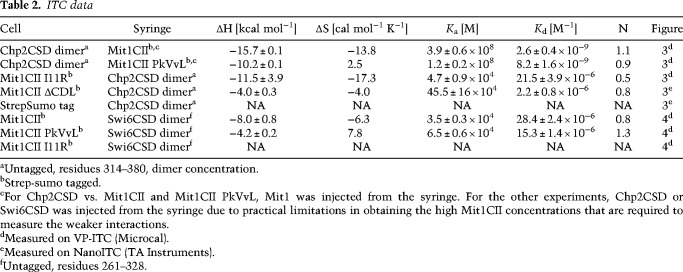
ITC data

We decided to mutate the central isoleucine to arginine to disrupt CkIvV binding to the dimerization groove and assess its contribution to the Chp2–Mit1 interaction. The Mit1I11R mutation in the CkIvV motif lead to a strong reduction in observable binding energy (Table 2; Fig. 3A) and resulted in a *K*_d_ of 21.5 µM, three to four orders of magnitude higher than the intact interface. A smaller drop in affinity to 2.2 µM is observed for the removal of the CDL (Table 2; Fig. 3A). Thus, CDL as well as CkIvV-mediated interactions make critically important contributions to the Chp2**–**Mit1 interaction interface. We conclude that the extensive contact surface observed in the crystal structure is required for providing high-affinity binding between Chp2 and Mit1.

### Disturbing the Chp2–Mit1 interaction abolishes transcriptional gene silencing

To test the functional significance of an intact Chp2**–**Mit1 interface we replaced the endogenous copy of Mit1 with Mit1 mutant versions targeting the CkIvV motif, the linker region and the CDL domain. Subjecting these strains to growth assays on selective medium revealed that disruptive mutations in any part of the Chp2**–**Mit1 interface lead to alleviated gene silencing comparable to the deletion of Chp2 or to the deletion of the Mit1 N terminus (Fig. 3B; Supplemental Fig. S3). Furthermore, we observed that heterochromatic transcript levels in selected mutants increased to similar levels as observed in *mit1ΔN* (Fig. 3B,C). These results further highlight the importance of an intact interface between Chp2 and Mit1 for appropriate gene silencing in heterochromatic regions.

The silencing assays, together with the structural and biophysical data, suggest that recruitment of Mit1 to heterochromatin is severely impaired when the Chp2**–**Mit1 interface is missing or partially disrupted. We therefore used chromatin immunoprecipitation (ChIP) at *dh* repeats against *mit1-13myc* wild-type and *mit1-13myc* interface mutants to test Mit1 recruitment to heterochromatin directly (Fig. 3D). These experiments show a complete loss of Mit1 upon deletion of the complete Mit1CII or upon disruption of the CkIvV motif by the I11R mutation, demonstrating that Mit1 association with heterochromatic sequences relies critically on the intact Chp2**–**Mit1 interaction. ChIP against the histone H3K14 acetyl mark and against RNA polymerase II reveals that loss of Mit1 correlates with the accumulation of H3K14ac and increased RNA polymerase II occupancy. Thus, disrupting the Chp2–Mit1 interface leads to the same loss of transcriptional gene silencing at heterochromatic repeats as observed for SHREC mutants (Sugiyama et al. 2007; Motamedi et al. 2008).

### Swi6 binds the Mit1 CkIvV motif, but lacks further interaction surface

**Figure 4.**
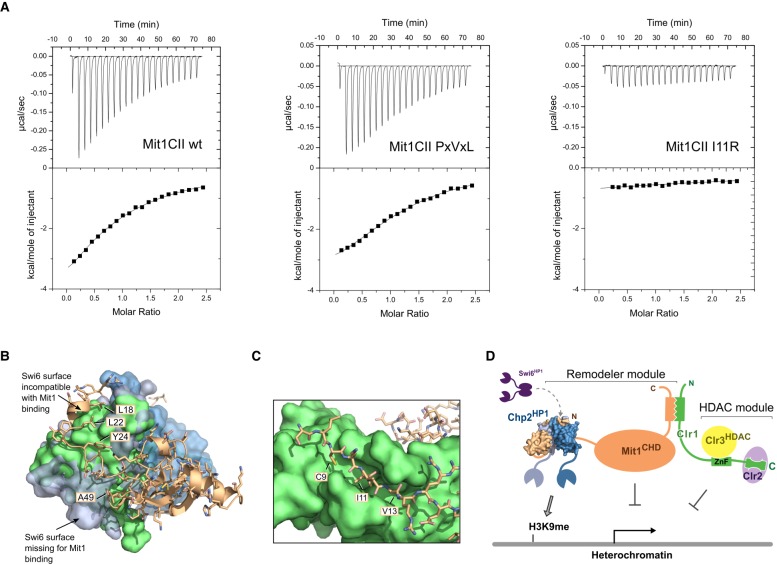
The Mit1 CkIvV motif also binds Swi6. (*A*) Heat rates and fit for ITC experiment where Swi6CSD is injected into a solution of Mit1CII. (*B*) Surface representation of Swi6 and Chp2 superimposed as in Supplemental Figure S4A shows that critical interactions in the extended interaction interface of Mit1 are blocked or are missing on the Swi6 surface. (*C*) Model of the Mit1 CkIvV motif in the CSD dimerization groove of Swi6. (*D*) The high-affinity Chp2–Mit1 interaction uncouples Mit1/SHREC recruitment to H3K9 methyl marks from Swi6. Schematic representation of the SHREC complex with the Chp2–Mit1 structure shown in surface representation summarizing the current structural knowledge on the complex (Job et al. 2016).

Several lines of evidence have implicated Swi6 in SHREC recruitment, but the mechanisms of this recruitment have remained elusive (Yamada et al. 2005; Sadaie et al. 2008; Fischer et al. 2009). Since Chp2 shows strong affinity for the PxVxL substitution it is conceivable that reversely Swi6 is able to bind the CkIvV motif. Indeed, we obtained a binding curve with a *K*_d_ of 28 µM for Swi6CSD binding to wild type Mit1CII (Table 2; Fig. 4A). Binding to the Mit1 PxVxL substitution mutant shows a tighter interaction with a *K*_d_ of 15 µM, while the Mit1 I11R mutation completely abolished the interaction and thereby corroborates the CkIvV motif as the site of interaction with Swi6. These observations reveal a complex interplay between the two HP1 proteins for Mit1, where a high-affinity interaction provides specificity for Chp2, while Swi6 has the potential to bind to the same region with low affinity.

The reason for Swi6's inability to bind Mit1 with high affinity can be rationalized by comparing the Chp2CSD dimer in the Chp2–Mit1 complex structure with the crystal structure of the free Swi6CSD dimer (Cowieson et al. 2000). They superimpose with a RMSD of 0.85 Å for Cα atoms and confirm the expected very close structural similarity (Supplemental Fig. S4A). Nevertheless, a striking difference between the two structures is observed in the folding of the N terminus where Chp2 shows seven structured residues more than Swi6, which form a loop that packs against the C-terminal β-strand and the following α-helix and thereby provide significantly more bulk to the Chp2 CDS (Fig. 4B). We also observe a molecule of hexanediol that is deeply buried by the Chp2 N terminus, and we speculate that it substitutes for one or two N-terminal residues by which our CSD construct is too short. The particular folding of the N terminus is likely to be independent of Mit1 binding, since it is identical between both CSDs even though only one is bound to Mit1 (Supplemental Fig. S4B). It is, however, noteworthy that the trajectory of the N-terminal loop differs around residue Q320 by a shift of up to 1.5 Å, which is probably due to the methyl-π interaction of Chp2F319 with Mit1A49 (Supplemental Fig. S4B). The Chp2 N terminus thus provides a unique binding surface for Mit1 that contributes to extension of the interaction interface when compared with Swi6.

Superposition of the surfaces of Chp2 and Swi6 also shows that the short helix in the linker region of Mit1 around residues 18–24 would collide with the surface of Swi6 (Fig. 4B). Furthermore, this surface exposes a hydrophobic patch in Chp2, while Swi6 forms an intramolecular salt bridge in the equivalent position, which disfavors accommodation of the hydrophobic stretch of Mit1 residues from L18 to L22. When analyzed in detail it is clear that the Swi6 sequence differs from Chp2 in several other key positions that are important for binding Mit1. These differences are conserved between Chp2 and Swi6 proteins of the *Schizosaccharomycetes* family (Supplemental Fig. S4C). Conversely, the N-terminal domain architecture of Mit1 proteins is also conserved, particularly in key hydrogen bonding positions like Y25 (Supplemental Fig. S4D), supporting the notion that the Chp2–Mit1 interaction is conserved across the *Schizosaccharomycetes* clade. Despite the differences between the Chp2 and Swi6 proteins, the canonical binding groove formed by the CSD dimerization interface of Swi6 can easily accommodate the CkIvV motif of Mit1 (Fig. 4C), which is entirely consistent with the ITC data.

## Discussion

### The unique structure of an HP1–client interface

The results presented here establish the mechanistic details of how the HP1 protein Chp2 interacts with the chromatin remodeler Mit1. The unique interaction between the two proteins combines binding in the canonical PxVxL binding groove at the dimerization interface of HP1 proteins with an extensive additional interaction interface created by wrapping the N-terminal domain of Mit1 around one of the Chp2CSDs. This elaborate interaction results in a low nanomolar dissociation constant and renders the Chp2–Mit1 interface highly specific, thereby explaining the exclusive client selectivity of Chp2 observed in previous experiments (Motamedi et al. 2008; Fischer et al. 2009), and why Chp2 is a de facto constitutive subunit of SHREC. The dedicated interface thereby uncouples SHREC's recruitment to heterochromatin from Swi6 and enables the cell to specifically regulate SHREC levels at heterochromatin loci through fine tuning expression levels of Chp2 (Fig. 4C).

The gain in specificity can be attributed to sequence differences in key surface residues that are involved in shape complementarity and hydrogen bonding networks between Chp2 and Mit1. The extended interaction interface is provided by the linker and the well folded CDL domain in Mit1, which has been missed so far by sequence comparison. It clearly resembles a CD and demonstrates a further use of this versatile fold in protein–protein interactions. The yeast-two-hybrid results suggest involvement of Chp2 domains other than the CSD. To our knowledge, the currently available evidence does not rule out the possibility of an even more extensive interface between Mit1 and Chp2.

With the CkIvV sequence bound to the CSD dimer interface the Chp2–Mit1 structure reveals a remarkably degenerate equivalent of the PxVxL motif that is not recognized by motif searches. Sequence comparison of Mit1 in *Schizosaccharomycetes* species indicates that the cysteine residue in the −2 position is poorly conserved and that it can accommodate lysine and arginine (Supplemental Fig. S4D). The 0 and +2 positions on the other hand are confined to the hydrophobic side chains valine or isoleucine. This suggests that the motif in the Mit1 context corresponds to xx[IV]x[IV]. Interestingly, Swi6 is also able to bind this motif, though with an affinity that is weaker than for *bona fide* Swi6 clients (Isaac et al. 2017) or the Mit1 PxVxL substitution.

### Regulation of Mit1

Our findings are consistent with previous observations that Mit1 and Chp2 are closely associated both functionally and physically (Motamedi et al. 2008; Fischer et al. 2009; Job et al. 2016). The identification of the physical interface between Chp2 and Mit1 fleshes out the connectivity of subunits in the SHREC complex and establishes the nucleosome remodeler Mit1 as the connecting subunit between Chp2 and Clr1, which interacts with the C-terminal domain of Mit1 (Fig. 4D). We further demonstrate that the repressive function of Mit1 and Chp2 depends strongly on their high-affinity interaction, which serves to recruit Mit1 to heterochromatic loci. Mit1 also harbors a PHD domain that binds histone H3 and potentially activating marks (Creamer et al. 2014), and it also interacts with Clr1, which connects Mit1 and Chp2 to the HDAC module of SHREC (Job et al. 2016). It is likely to be an equilibrium between these factors that controls Mit1's activity in transcriptional gene silencing. The identification of the Chp2–Mit1 interface and its atomic structure provides critical information to elucidate the recruitment mechanisms of SHREC and to inform on mechanisms governing the family of NuRD complexes.

### Role of Chp2 and Swi6 HP1 isoforms

The Chp2–Mit1 high-affinity interaction described here explains why Chp2 is almost exclusively found in complex with components of the SHREC complex (Motamedi et al. 2008). On the other hand, several reports have shown that Swi6 is implicated in SHREC recruitment (Yamada et al. 2005; Sugiyama et al. 2007; Sadaie et al. 2008; Fischer et al. 2009). Our finding that Swi6 can also bind to the Mit1 N terminus provides an explanation that reconciles these results. The high abundance of Swi6 makes it plausible that it significantly contributes to Mit1 and SHREC recruitment despite its lower affinity for Mit1. Recent results also suggest that Swi6 interacts with Clr3, the HDAC of the SHREC complex, which might further contribute toward SHREC recruitment (Isaac et al. 2017). However, we show here that Swi6 alone cannot support Mit1 function in CDL mutants, and Swi6 overexpression only partially complements loss of Chp2 (Sadaie et al. 2008). These observations suggest that the fission yeast heterochromatin system relies on the specific aspects of the Chp2–Mit1 interaction. One possibility is that the high-affinity interaction permits Chp2 to efficiently recruit a very low abundance complex like SHREC to the H3K9 methyl mark without raising HP1 protein concentrations to levels that would disrupt the equilibrium between HP1 proteins, H3K9 methyl marks and HP1 client proteins.

Sequence comparison suggests that the mechanism of the Chp2–Mit1 interaction is conserved in the *Schizosacharomycetes* family, and how HP1 proteins interact with nucleosome remodelers remains an interesting subject. Human HP1 proteins have been found to associate with the Mit1 homologue CHD4 (Vermeulen et al. 2010), and recent evidence has revealed that human HP1 and CHD4 form a complex with the DNA-binding factor ADNP to regulate lineage-specific gene expression in mouse embryonic stem cells (Ostapcuk et al. 2018).

The findings presented here provide key information to study fundamental principles of how HP1 proteins and the chromatin remodeling machinery interact to regulate gene expression.

## Materials and methods

### Pull-down experiments

All sequences were cloned into YFP-containing vectors of the Multibac system (Bieniossek et al. 2009) using Gibson cloning (Gibson et al. 2008). Mit1 constructs were cloned into acceptor vectors as N-terminal fusions of OneStrep-MBP tags or OneStrep Sumostar tags, followed by a T7 tag (Novagen) and a TEV cleavage site. Chp2 and the Clr1MID construct (included to stabilize the Mit1 C terminus) (Job et al. 2016) were cloned into donor vectors as N-terminal fusions to HisSUMO followed by T7 tags. The HisSUMO tag is cleaved off in the cells during expression by endogenous SUMO proteases, yielding protein carrying only the T7 tag. Donor and acceptor vectors were combined by Cre recombination for coexpression as needed. Proteins were expressed in transfected Sf9 insect cells in adhesion culture in six well plates with 3 × 10^6^ cells per well in 3 mL of media (Amimed) at 27°C. Cells were harvested by aspirating the media when all cells were expressing YFP and frozen (−80°C) until further use. Cells were scraped from the plate surface under the addition of pull-down buffer (400 mM KCl, 100 mH Tris at pH 7.5, 2 mM Mg-acetate, 5 mM β-mercaptoethanol) supplemented with 0.1% NP40 and protease inhibitors, cell debris was removed by centrifugation and the supernatant was incubated with StrepTactin (IBA) beads for 1 h at 4°C with agitation. After incubation, unbound material was removed, the beads were washed five times with pull-down buffer, and bound proteins were eluted with elution buffer (pull-down buffer + 5 mM desthiobiotin).

### Western blotting

Samples were run on Bis-Tris gels and transferred to nitrocellulose membrane (Biorad). Proteins were detected by Western blotting using antibody against either tag or protein of interest, followed by incubation with secondary antibody labeled with the DyLight system and scanning with the Odyssey Imaging System (LI-COR).

### Yeast two-hybrid screening

Chp2 constructs were cloned in-frame to the Gal4 binding domain into the pGBKT7 vector, transformed into the Y2Hgold strain and selected on SD/-Trp medium. Mit1 construct-containing pGADT7 vectors were transformed into the Y187 strain and transformed cells were selected on SD/-Leu medium. Appropriate strains were mated and selected for on SD/-Trp/-Leu. To detect protein interactions, growth assays were performed at 30°C on SD/-Trp/-Leu/-His and SD/-Trp/-Leu/-His/-Ade medium.

### Protein expression and purification

All sequences were cloned into vectors of the MultiColi system (Bieniossek et al. 2012) using Gibson cloning (Gibson et al. 2008). All constructs for individual expression were cloned as N-terminal fusions to a Strep-SUMO-tag into the pACE1 vector, while for coexpression the chromo shadow domain of Chp2 was cloned without tag into pACE2.

Proteins were expressed in BL21(DE3) cells that carried an additional plasmid encoding the trigger factor chaperone (Nishihara et al. 2000) or Rosetta (DE3) (Novagen). Cells were grown to log phase at 37°C, cooled on ice, and induced with 0.4 mM IPTG and 0.5 mg/mL l-Arabinose before incubation for 18 h at 18°C. Pellets were resuspended in phosphate buffered saline (PBS) supplemented with protease inhibitors and frozen at −80°C until further usage. For purification, cell pellets were thawed in purification buffer (400 mM KCl, 50 mM Tris at pH 7.5, 5 mM β-mercaptoethanol) supplemented with protease inhibitors and cells were ruptured using either sonication or the Emulsiflex homogenizer. Cell debris was removed by centrifugation and the supernatant was bound to a 5-mL Streptactin column (Qiagen). After washing with purification buffer, tagged protein was eluted with purification buffer + 5 mM desthiobiotin except for Swi6, which was subjected to overnight on column cleavage with Ulp1 protease. Chp2–Mit1 complex intended for crystallization was treated with Ulp1 protease overnight. The Mit1 constructs intended for ITC were left with the Strep-SUMO-tag on for stabilization. Chp2 was treated with Ulp1, dialyzed into low salt buffer (200 mM KCl, 50 mM Tris at pH 7.5, 5 mM β-mercaptoethanol) and further purified using cation-exchange chromatography (MonoS, GE Healthcare) where it eluted as a single peak during a salt gradient from 200–1000 mM NaCl. All other proteins were subjected to size exclusion chromatography (Superdex 75, GE Healthcare) in purification buffer. Peak fractions were pooled and concentrated as needed (Amicon Ultra-4 10 kDa, Millipore).

### Limited proteolysis

Thermolysin was prepared as a 1 mg/mL stock solution in thermolysin buffer (10 mM Tris-HCl at pH 8.0, 200 mM NaCl, 2 mM CaCl_2_, 5% glycerol). Twelve reactions containing 5 µg purified Mit1(1-300)-Chp2CSD complex each were prepared and incubated at room temperature for 30 min with different amounts of thermolysin, ranging from ratios of 2:5 to 1:5120 protein:thermolysin. Reactions were quenched by adding 4× SDS loading dye +50 mM EDTA and the formation of stable fragments was analyzed on Bis-Tris-gels. The ratio producing the largest amount of stable fragments was scaled up to 600 µg of protein complex and purified using size exclusion chromatography (Superdex 75, GE Healthcare). Peak fractions were pooled, dialyzed against 0.1% acetic acid for 3 h, and lyophilized. Peptide masses were determined by MS/MS Maldi-TOF analysis at the Functional Genomics Center Zurich (FGCZ).

### Crystallization and structure processing

Mit1(1-81)–Chp2(316-380) were grown at 18°C as 1-µL hanging drops in a 1:1 ratio of protein to reservoir solution (2.4 M sodium malonate at pH 7, 3% 1,6-hexanediol). Crystals were flash frozen in liquid nitrogen and datasets were collected at beamline PXIII at the SLS, PSI Villigen. Data were processed with XDS (Kabsch 2010) and scaled with Aimless (Evans and Murshudov 2013), and the structure was solved by molecular replacement using Swi6 as a model (PDB ID 1E0B) with Phaser (McCoy et al. 2007). The model was built in Coot (Emsley and Cowtan 2004) and refined with Phenix (Emsley and Cowtan 2004; Adams et al. 2010). Pymol (Schrödinger) was used for the preparation of figures.

### ITC

ITC experiments were performed at 25°C using a VP-ITC (Microcal) and a Nano ITC calorimeter (TA instruments). All proteins used were dialyzed overnight against ITC buffer (400 mM NaCl, 50 mM Tris-HCl at pH 7.5, 5 mM β-mercaptoethanol) prior to experiments. Ten microliters of Chp2 or Swi6 at 100 to 500 µM was injected in 200-sec time intervals into the cell holding Mit1 constructs at concentrations between 5 and 50 µM. After subtracting heat enthalpies for titrations of the respective proteins into buffer, the ITC data were analyzed with OriginLab (Figs. 3, 4) or NanoAnalyze Data Analysis software (TA instruments)(Fig. 3, Mit1CIIΔCDL). Protein concentrations were determined by absorbance at 280 nm.

### Generation of *S. pombe* strains

*S. pombe* strains were grown and manipulated as previously described (Job et al. 2016), and strains used in this study can be found in Supplemental Table S1. For crosses, tetrad dissection analysis was used with plating on selective media and followed by PCR to check the obtained genetic backgrounds.

A strain in which endogenous Mit1 was replaced by the rpl42-natMX cassette (*mit1::rpl42-natMX*) was obtained by transforming wild-type yeast with a DNA fragment (generated by restriction digest of the respective plasmid) containing the rpl42-natMX cassette flanked on either side by 500 bp of respective sequence found in the 5′- and 3′-UTR of Mit1, making use of homology recombination for insertion into the genome (Bähler et al. 1998; Fennessy et al. 2014). The strain was selected by rounds of positive and negative selection using the cycloheximide sensitivity conferred by rpl42^+^ and the nourseothricin (clonNAT) resistance conferred by natMX.

Strains carrying full-length Mit1 C-terminally tagged with 13 copies of the *c-myc* epitope (13myc) at the endogenous locus or truncations or mutations thereof were generated by replacing the rpl42-natMX cassette in the *mit1::rpl42-natMX* strain mentioned before with 13myc-tagged constructs of choice, by transformation with respective DNA fragments and homology recombination.

For silencing assays, strains were generated by crossing with a *otr1::ura4*+ reporter strain. Silencing of *ura4*^+^ renders cells auxotrophic for uracil and resistant to 5-fluoroorotic acid (5-*FOA*).

### Silencing assays *S. pombe*

*otr1::ura4+* reporter strains were grown overnight at 30°C in YES to a density of 5 × 10^6^ cells/mL. Cells were pelleted by centrifugation and resuspended in water. Tenfold serial dilutions were spotted onto PMG + adenine + leucine + uracil (nonselective), PMG + adenine + leucine (−ura), PMG + adenine + leucine + 100 mg/L uracil + 2 mg/mL 5-FOA (FOA), with 1 × 10^4^ cells in the highest-density spots.

### Small-scale protein extraction from *S. pombe*

Strains were grown in YES to a density of 5 × 10^6^ cells/mL, and 6 × 10^7^ cells per strain were pelleted by centrifugation and resuspended in 300 µL of 4× cOmplete, EDTA-free (Roche), and 2 mM PMSF on ice. Samples were mixed with 300 µL of cold NaOH (0.6 M), incubated for 10 min on ice, and spun down. The supernatant was removed, and the pellet was resuspended in 70 µL of gel loading dye supplemented with 2× cOmplete, 1 mM PMSF, and 4% βme before boiling for 3 min at 98°C.

### Coimmunoprecipitation from *S. pombe*

Strains were grown in YES to a density of 5 × 10^6^ cells/mL; pelleted by centrifugation; resuspended in cold PBS supplemented with 1× cOmplete, EDTA-free (Roche), 1 mM PMSF, and 10 mM DTT; and frozen in liquid nitrogen. Then, 150 × 10^6^ cells were thawed in lysis buffer (50 mM HEPES at pH 7.5, 300 mM NaCl, 1 mM EDTA, 5 mM CHAPS, 10 mM DTT, 1 mM PMSF, 1× cOmplete [Roche]), added to glass beads, and subjected to two 20-sec runs in a beadbeater for cell disruption. Debris was removed by centrifugation, and the lysate was incubated with Myc-Trap_MA resin (Chromotek) for 1 h with agitation. After washing, bound proteins were eluted by adding 30 µL of gel loading dye +10 mM DTT and boiling for 5 min.

### ChIP

Cells for ChIP were grown in 50 mL of YES medium to a density of 1.2 × 10^7^ cells/mL. For RNA polymerase II and H3K14ac, cells were fixed in 1% formaldehyde for 30 min. For Mit1-13myc, a dual-cross-linking approach was employed as previously described (Tian et al. 2012). Briefly, cells were incubated for 2 h at 18°C, pelleted, resuspended in 5 mL of PBS, and cross-linked at room temperature with 1.5 mM ethylene glycol bis-succinimidyl succinate (EGS, Thermo Scientific). After 30 min of incubation, cells were cross-linked in 1% formaldehyde. Cells were resuspended in ChIP Buffer (50 mM HEPES/KOH at pH 7.6, 150 mM NaCl, 1 mM EDTA, 1% Triton X-100, 0.1% Na-Deoxycholate, 1 mM PMSF, 5 mM Na-Butyrate, 1× cOmplete [Roche]) and lysed by beadbeating. Chromatin was enriched by centrifugation and sonicated for 15 min (30 sec/30 sec on/off) in a Bioruptor Pico. Fifty microliters of sheared soluble chromatin was diluted with 450 µL of ChIP Buffer, mixed with 1 µg of antibody (H3K14ac [ab52946], RNA PolII [ab817], and Myc [mAb #2276]), and incubated for 2 h, followed by 45 min of incubation with Protein A/G magnetic beads. The bead/protein complex was washed three times with ChIP Buffer; once with 50 mM HEPES/KOH (pH 7.6), 500 mM NaCl, and 1 mM EDTA; once with 5 mM Tris-Cl (pH 8), 250 mM LiCl, 0.5% Triton X-100, 0.5% Na-Deoxycholate, and 0.05% Tween 20; and once with TE (10 mM Tris-Cl at pH 8, 1 mM EDTA). The protein–DNA complex was eluted in 50 mM Tris-Cl (pH 8), 10 mM EDTA, and 1% SDS for 15 min at 65°C, and the cross-linking was reversed overnight at 65°C. The samples were then treated with proteinase K and DNA was purified by phenol-chloroform. qPCR was performed using primers given in Supplemental Table S2, and *act1*+ was used as internal control.

### RT-qPCR

Strains were grown in YES to a density of 5 × 10^6^ cells/mL, pelleted by centrifugation, and washed with water. RNA isolation was performed with Trizol extraction followed by phenol/chloroform extraction and ethanol precipitation. DNA was then further removed by DNaseI treatment and phenol/chloroform extraction followed by ethanol precipitation. cDNA was generated with EvoScript Universal cDNA Master kit (Roche), and qPCR was performed using SYBR Green I Master kit on LightCycler 480 instrument (Roche). qPCR primers used in this study are listed in Supplemental Table S2. Data were analyzed using the ΔΔ*C*_t_ method (Yuan et al. 2006).

### Accession codes

Coordinates and structure factors have been deposited in the Protein Data Bank under accession code 6FTO.

## Supplementary Material

Supplemental Material
