# Supplementary material for: Transcriptional gene silencing requires dedicated interaction between HP1 protein Chp2 and chromatin remodeler Mit1
Source: Genes Dev. 2019 May 1;33(9-10):565–77. doi: 10.1101/gad.320440.118 (PMC6499331; doi:10.1101/gad.320440.118)
Supplement: Supplemental Material [file supp_33_9-10_565__index.html]

Transcriptional gene silencing requires dedicated interaction between HP1 protein Chp2 and chromatin remodeler Mit1 — Supplemental Material 

# Transcriptional gene silencing requires dedicated interaction between HP1 protein Chp2 and chromatin remodeler Mit1

## Supplemental Material

- Supplemental\_Information.pdf
