# Supplementary material for: Transcriptional gene silencing requires dedicated interaction between HP1 protein Chp2 and chromatin remodeler Mit1
Source: Genes Dev. 2019 May 1;33(9-10):565–77. doi: 10.1101/gad.320440.118 (PMC6499331; doi:10.1101/gad.320440.118)
Supplement: Supplemental Material [file supp_gad.320440.118_Supplemental_Information_1.pdf]

Supplemental Information

Transcriptional gene silencing requires dedicated interaction between HP1 protein Chp2 and chromatin remodeler Mit1

Karoline Leopold[1], Alessandro Stirpe[1], Thomas Schalch[1,2%]

[1] Department of Molecular Biology, Faculty of Science, Sciences III, University of Geneva, CH-1211 Geneva 4, Switzerland

[2] Leicester Institute for Structural and Chemical Biology, Department of Molecular and Cell Biology, University of Leicester, Leicester, LE1 9HN, UK

Supplemental Figures S1-S4
Supplemental Tables S1-2

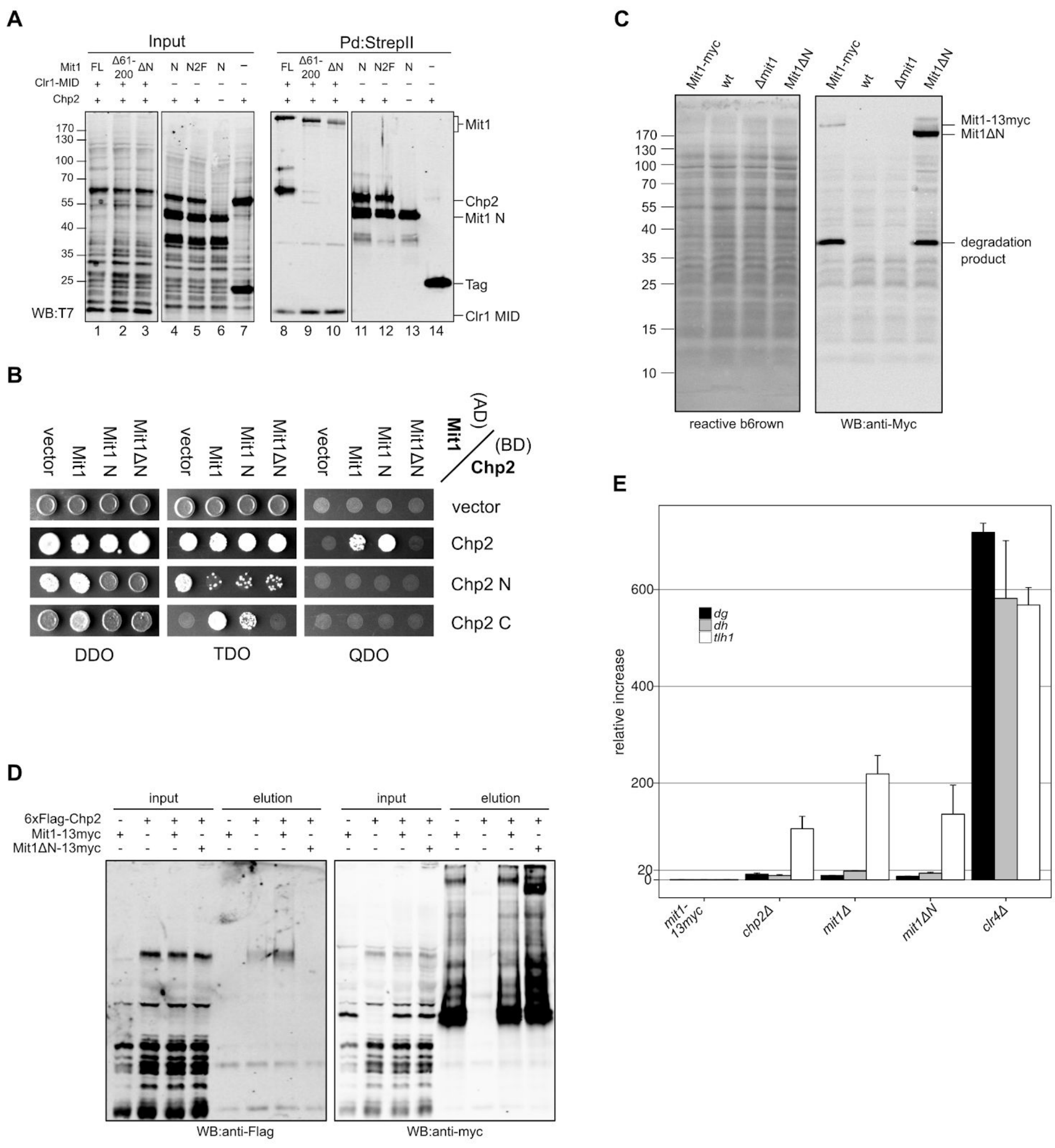

Figure S1: Expression levels of Mit1 constructs. (A) Pull-down experiments with a co-expressed ternary complex of Chp2, Clr1's Mit1 interacting domain (MID) and StrepII-tagged Mit1 fragments from insect cells. N2F corresponds to combined PxVxL motif mutants V20F and V72F. (B) Yeast-2-hybrid interaction screen between Mit1 and Chp2 fragments. DDO: double dropout medium SD/-Trp/-Leu; TDO: triple dropout medium; SD/-Trp/-Leu/-His; QDO: quadruple dropout medium SD/-Trp/-Leu/-His/-Ade (C) Western blot with anti-Myc antibody on *S. pombe* whole cell lysates for indicated strains. Reactive brown, a non-specific protein stain, shows sample loading. (D) Whole size blot for co-IP experiment in Fig. 1D. (E) Rescaled version of Fig. 1D including *clr4Δ*.

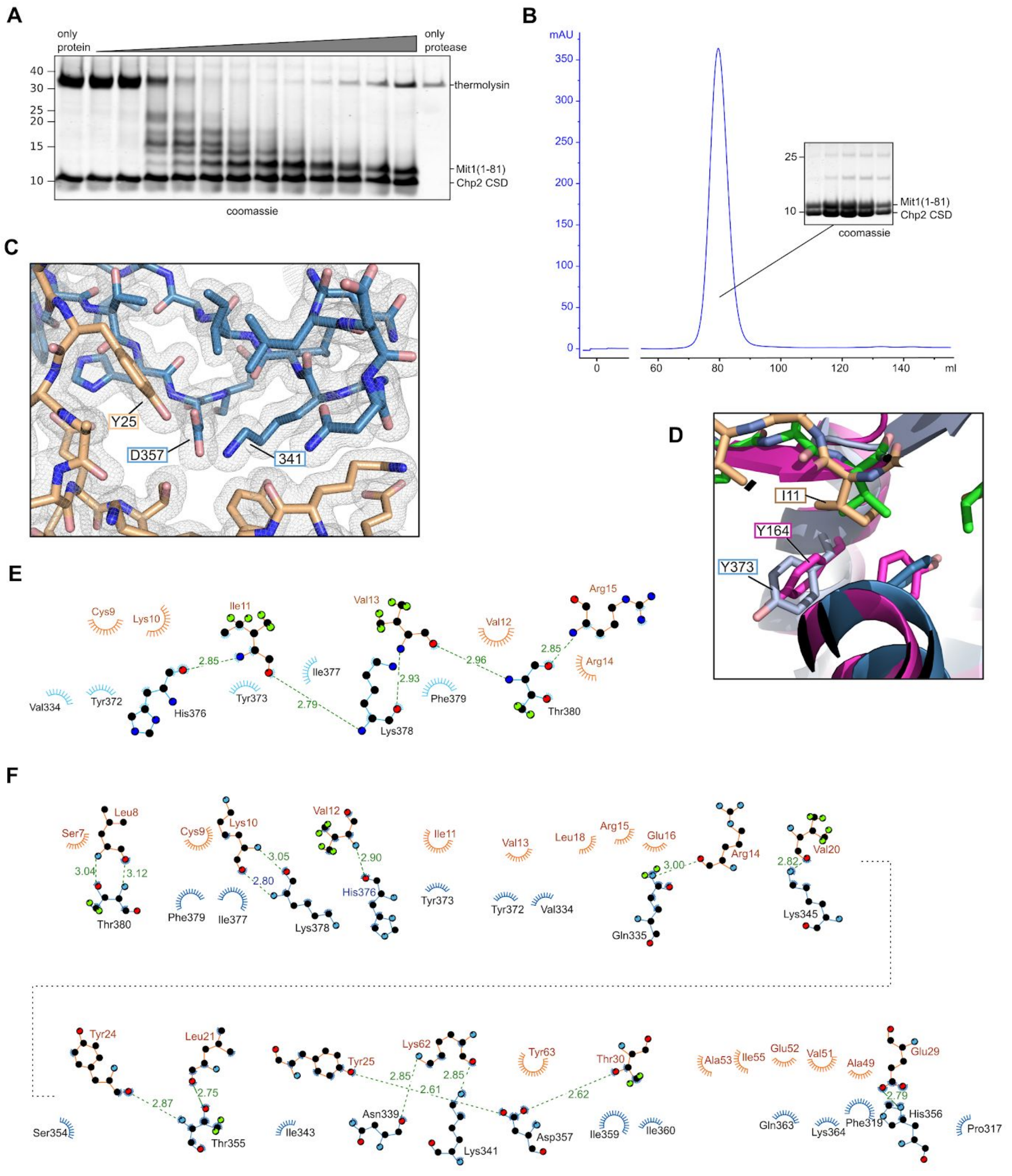

Figure S2: Limited proteolysis reveals minimal Chp2–Mit1 complex. (A) SDS PAGE gel of limited proteolysis with thermolysin on the Chp2–Mit1(1-299) complex. (B) The Chp2–Mit1(1-81) complex elutes as a single peak from SEC. (C) Electron density (gray) for interface between Chp2 CSD and the Mit1 linker segment around residue Y25 contoured at 1.5 σ. (D) Superposition of Chp2–Mit1 (blue-orange) onto the HP1–CAF-1 (pink-green, PDBID:1S4Z) complex suggests how rotation of the Chp2Y373 side chain at the central position of the dimerization groove can accommodate the extra methyl group of Chp2I11. (E) LigPlot+ interaction diagram for Mit1 (orange) and Chp2 CDS1 (light blue). (F) LigPlot+ interaction diagram for Mit1 (orange) and Chp2 CSD2 (dark blue)

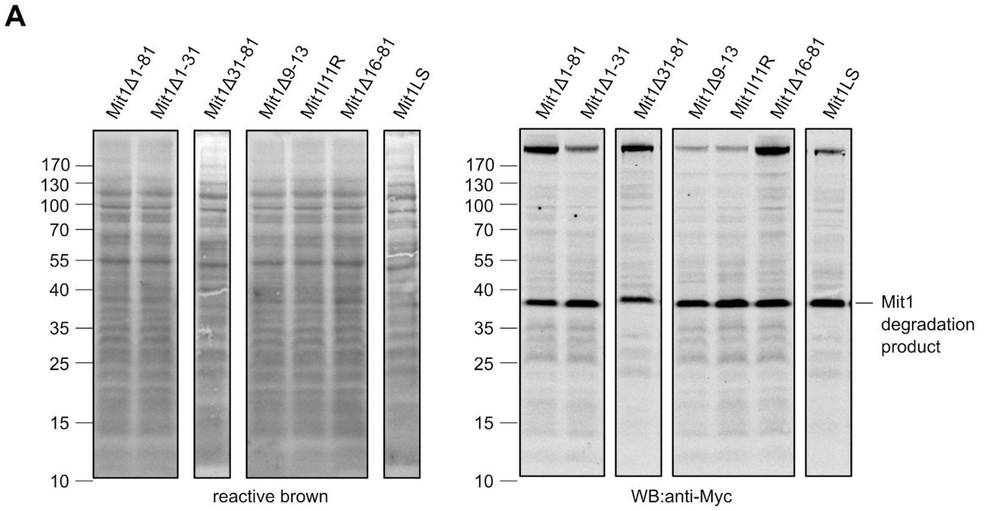

Figure S3: . Protein levels of Mit1 mutants. (A) Western blot with anti-Myc antibody on S. pombe whole cell lysates for indicated strains. Reactive brown, a non-specific protein stain, shows sample loading. Lanes are cut from two simultaneously and identically performed Western blots.

Leopold_Fig. S4

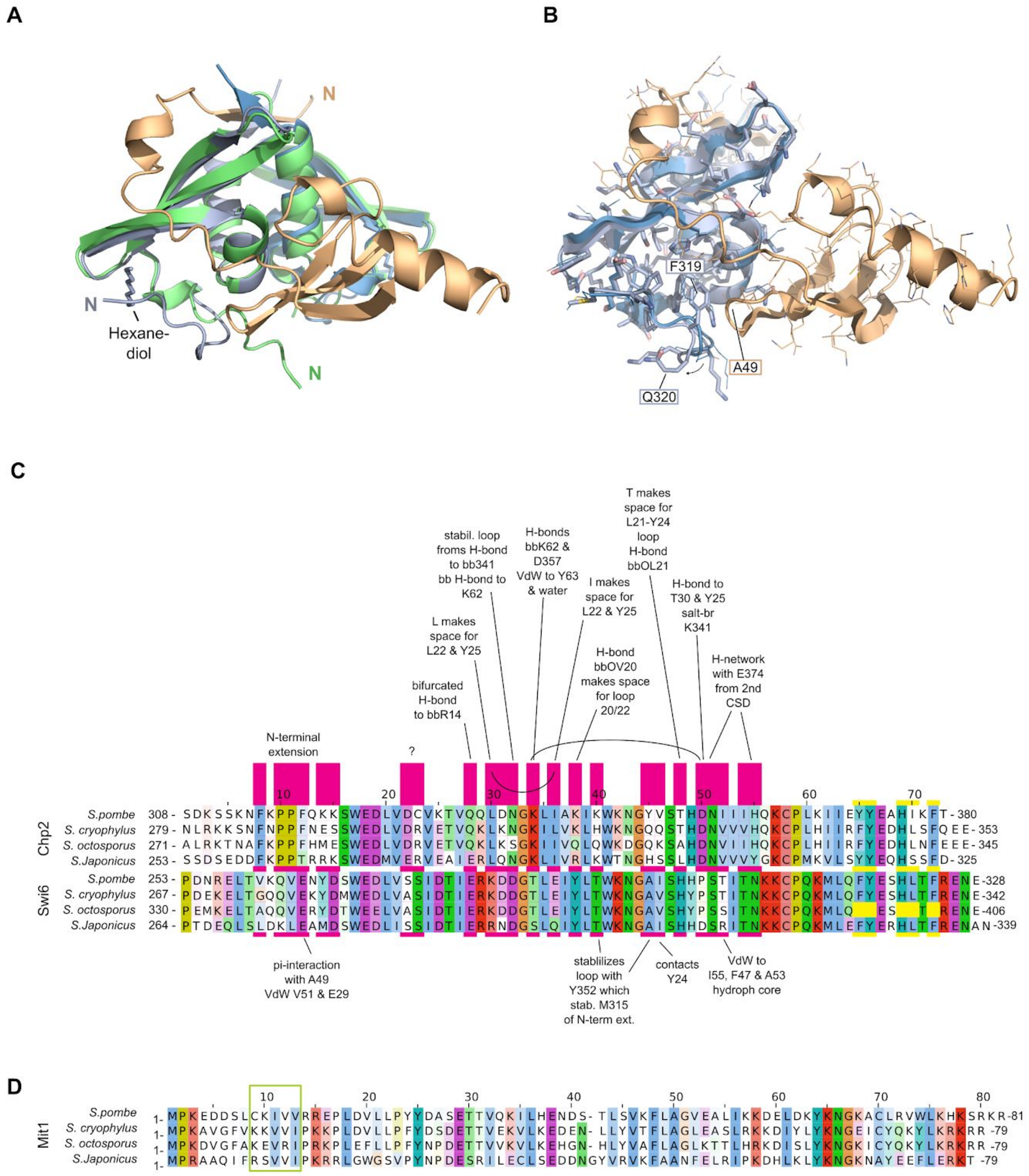

Figure S4: Chp2 and Swi6 sequence differences are conserved. (A) Swi6CSDs superimposed onto Chp2CSDs with Cα RMSD of 0.85 Å (Chp2 and Mit1 colored as in Fig. 2, Swi6CSD in green, PDBID: 1E0B). Note divergence of N-termini. (B) Superposition of CSD2 (darker blue) onto CSD1 (lighter blue), which is bound to Mit1, with Cα RMSD of 0.3 Å. Side chains of the the Mit1-bound CSD1 are shown in stick representation. (C) Comparison of conserved positions in Swi6 and Chp2 CSD alignments across *Schizosaccharomycetes* species. Annotations describe observations with respect to complex formation with Mit1 (bb=back bone). (D) Multiple sequence alignment of N-terminal Mit1 domains. Green box marks CkIvV motif.

Supplemental Tables

Table S1. Strains used in this study

Name	Genotype	Figure	Source
S.0DM	h- rpl42-P56Q(cyhR) ade6M210 ura4-D18 mit1-13myc::KanMX6	1D, 1F	this study
S.0JP	h+ chp2::3xFLAG-(x2)-chp2+ leu1-32 ura4D18 his7-366 ade6DN/N	1D	Nakayama lab
S.0L0	h- ade6M210 ura4-D18 mit1::mit1ΔN-13myc_KanMX6 chp2::3xFLAG-(x2)-chp2+	1D	this study
S.0L1	h- ade6M214 ura4-D18 mit1::mit1-13myc_KanMX6 chp2::3xFLAG-(x2)-chp2+	1D	this study
S.0DY	h+ ura4DS/E otr1R::ura4+ leu1-32 ade6-216	1E, 3C	(Sugiyama et al. 2007)
S.0E0	h+ ade6-M216 ura4-DS/E mit1::rpl42-natMX6(Δmit1) otr1R::ura4 leu1-32	1E, 3C	this study
S.0E2	h+ ade-M216 ura4-D18 mit1ΔN-13myc::KanMX6 leu1-32 otr1R::ura4	1E	this study
S.0EH	h- ade6-M216 ura4-D18 mit1-13myc::KanMX6 otr1R::ura4	1E, 3C	this study
S.0D9	h- rpl42-P56Q(cyhR) ade6-M210 ura4-D18 mit1ΔN-13myc::KanMX6	1F, 3D	this study
S.05O	h- 972/WT	1F	Simanis lab
PY4281	h- chp2Δ::KanMX6 ade6-210 arg3-D4 his3-D1 leu1-32 ura4-D18	1F	(Job et al. 2016)
S.0D1	h- rpl42-P56Q(cyhR) ade6M210 ura4-D18 mit1::rpl42-natR(Δmit1)	1F	this study
S.0H0	h+ clr4Δ::KanMX6 ade6M210? ura4-D18	1F	this study
S.0FZ	h+ chp2Δ::KanMX6 otr1R::ura4+ leu1-32 ade6-M210 ura4-D18	3C	this study
S.0II	h- mit1::mit1LS-13myc-KanMX6 ade6-210 ura4-D18 leu1-32 otr1R::ura4+	3C	this study
S.0IJ	h+ chp2::chp2-3xFlag-KanMX6 mit1::mit1Δ(31-81)-13myc-KanMX6 ade6-216 ura4-D18 leu1-32 otr1R::ura4+	3C	this study
S.0IL	h+ ade6M216 ura4-D18 mit1::mit1Δ(1-31)-13myc-KanMX6 otr1R::ura4+	3C	this study
S.0IN	h+ ade6M210 ura4-DS/E mit1::mit1Δ(16-81)-13myc-KanMX6 otr1R::ura4+	3C	this study
S.0IQ	h- ade6M210 ura4-D18 mit1::mit1I11R-13myc-KanMX6 otr1R::ura4+	3C	this study
S.0IU	h- ade6M216 ura4-DS/E mit1::mit1Δ(9-13)-13myc-KanMX6 otR1::ura4+	3C	this study
S.0IW	h+ ade6M216 ura4-D18 leu1-32 mit1::mit1Δ(1-81)-13myc-KanMX6 otR1::ura4+	3C	this study
S.0GQ	h- rpl42-P56Q (cyhR) ade6M210 ura4-D18 mit1::mit1LS	3D	this study
S.0GT	h- rpl42-P56Q (cyhR) ade6M210 ura4-D18 mit1::mit1Δ(31-81)	3D	this study
S.0GX	h- rpl42-P56Q (cyhR) ade6M210 ura4-D18 mit1::mit1Δ(1-81)	3D	this study

Table S2. Oligonucleotides

Name	Construct	Sequence	Use
O.1I0	tlh1_R	CTCCTTGGAAGAATTGCAAGCCTC	Fig. 1D, 3C
O.1HZ	tlh1_F	ATGGTCGTCGCTTCAGAAATTGC	Fig. 1D, 3C
O.1HY	cen-dg_R	TGCTTCACGGTATTTTTTGAAATC	Fig. 1D, 3C
O.1HX	cen-dg_F	AAGGAATGTGCCTCGTCAAATT	Fig. 1D, 3C
O.1HW	cen-dh_F	GTATTTGGATTCCATCGGTACTATGG	Fig. 1D, 3C, 3D
O.1HV	cen-dh_R	ACTACATCGACACAGAAAAGAAAACAA	Fig. 1D, 3C, 3D
O.1K8	ura4_RT-Rv1	GAAGACATTTCAGCCAAAAGCA	Fig. 1C, 3B
O.1K7	ura4_RT-Fw1	GGCCTCAAAGAAGTTGGTTTACC	Fig. 1C, 3B
O.10Z	act1_R	TCTTTTCCATATCATCCCAGTTG	Fig. 1F, 3D
O.10Y	act1_F	CTCAAAGCAAGCGTGGTATTT	Fig. 1F, 3D
